# Energy-Efficient and Variability-Resilient 11T SRAM Design Using Data-Aware Read–Write Assist (DARWA) Technique for Low-Power Applications

**DOI:** 10.3390/s23115095

**Published:** 2023-05-26

**Authors:** Sargunam Thirugnanam, Lim Way Soong, Chinnaraj Munirathina Prabhu, Ajay Kumar Singh

**Affiliations:** 1 School of Engineering and Computing, Manipal International University, Nilai 71800, Malaysia; 2 Faculty of Engineering and Technology, Multimedia University, Melaka 75450, Malaysia; 3 Electronics and Communication Engineering Department, NIIT University, Alwar 301705, India

**Keywords:** static random-access memory (SRAM), energy efficient, variability resilient, process variations, static noise margin, write ability, Monte Carlo simulation, low power

## Abstract

The need for power-efficient devices, such as smart sensor nodes, mobile devices, and portable digital gadgets, is markedly increasing and these devices are becoming commonly used in daily life. These devices continue to demand an energy-efficient cache memory designed on Static Random-Access Memory (SRAM) with enhanced speed, performance, and stability to perform on-chip data processing and faster computations. This paper presents an energy-efficient and variability-resilient 11T (E^2^VR11T) SRAM cell, which is designed with a novel Data-Aware Read–Write Assist (DARWA) technique. The E^2^VR11T cell comprises 11 transistors and operates with single-ended read and dynamic differential write circuits. The simulated results in a 45 nm CMOS technology exhibit 71.63% and 58.77% lower read energy than ST9T and LP10T and lower write energies of 28.25% and 51.79% against S8T and LP10T cells, respectively. The leakage power is reduced by 56.32% and 40.90% compared to ST9T and LP10T cells. The read static noise margin (RSNM) is improved by 1.94× and 0.18×, while the write noise margin (WNM) is improved by 19.57% and 8.70% against C6T and S8T cells. The variability investigation using the Monte Carlo simulation on 5000 samples highly validates the robustness and variability resilience of the proposed cell. The improved overall performance of the proposed E^2^VR11T cell makes it suitable for low-power applications.

## 1. Introduction

Low-power applications, namely smart sensor nodes, mobile applications, and portable digital gadgets, are becoming a vital part of human life. The significance of these low-power applications has influenced the simplification of the operation of most industries, ranging from military, healthcare, manufacturing, agriculture, travel and tourism, telecommunication, and transportation. The Internet of Things (IoT) revolution has impacted every home, every office, and every industry and has had a significant effect on every individual lifestyle. Low-power IoT sensor nodes are commonly used in smart cities, smart vehicles, smart buildings, smart agriculture, and smart homes. IoT applications will drive human experiences in the future [1], and these are normally connected to many portable and battery-operated gadgets using sensor nodes. The lower power consumption requirement is the most critical aspect of these IoT applications [2], which generally bridges the communication and connectivity to gather data between different nodes and base station for further data processing using wireless protocols [3]. Static Random-Access memory (SRAM) makes up a major portion of every low-power application. SRAM memory is also an everlasting and highly critical device for each and every low-power application and a common architecture among all the memory systems due to its performance [4]. High-speed cache memories are normally designed with SRAM.

Low-power applications require SRAM-based cache memory due to its low-power and high-performance features. There are many design techniques that have been proposed by researchers regarding energy-efficient SRAM cells with both merits and demerits [5,6]. In general, memory devices consume more power than the overall system power, as highlighted in the literature [6]. On average, 40–50% dynamic power is persistently consumed by SRAM memory devices in any IoT-based low-power system-on-chip (SoC) applications [7]. The on-chip SRAM cache consumes a major proportion of the total dynamic energy per operation. The earlier proposed cache memory, such as 2 Kb, 32 Kb, 60 Kb, and 128 Kb, designed with SRAM for biomedical microprocessors, have shown dynamic power consumption ranging from 47% to 63% [8]. Traditional cells, such as conventional 6T and standard 8T, have proven to be unsuitable for low-power applications. The everyday challenges of SRAM memory are the larger power dissipation, stability degradation, short channel effects (SCEs), and a higher leakage power and leakage current.

Many research projects have been carried out focusing on SRAM memory development by researchers in academia and different industries over many years [9,10,11,12,13,14,15] due to its performance and reliability. Researchers have proposed many different techniques and approaches to minimize the cache memory’s overall power [9,10,11,12,13,14,15]. The single-bit line operation [10,14], Schmitt trigger approach [9], loop cutting [11,14], differential operation [11,15], use of stack effect [11,15], power gating [12], decoupled read circuit [11,13,14,15], and lowering the supply voltage [12] are some of the main techniques applied for SRAM memory. The common technique employed in SRAM cells is to separate read and write operations, which significantly improves stability [16,17]. Despite having many different techniques applied to the design of SRAM memory, conventional and standard cells are not highly suitable for the latest and trending low-power applications [18,19] due to their consistent low-power requirements. Furthermore, as the technology transforms into a nanometer regime, the variation in process, voltage, and temperature (PVT) is a serious concern and adds to the existing challenges [13,15]. Therefore, it is essential to design an energy-efficient SRAM memory with variability resilience to address the above challenges and to exhibit the immunity to process variation with operational reliability. In this paper, an energy-efficient and variability-resilient SRAM memory is proposed and designed with the following salient features and novelties:

- A novel technique named Data-Aware Read–Write Assist (DARWA) is applied with single-ended read operation and dynamic differential write operation;
- The faster data switching happens when the latch circuit is totally disconnected at the nodes for write operations, which confirms lower dynamic power consumption due to lesser discharging at the bit lines (BL and BLB);
- The independent single-ended and separate read circuit performs a quicker read operation and hence reduces the read power consumption and enhances the read stability and overall read performance;
- The stack effect is introduced using tail transistors on both sides of the latch, which significantly minimizes the leakage power;
- The variability in the read, write, and hold modes is investigated and analyzed in detail to examine the resilience and robustness of the proposed cell using 5000 samples in Monte Carlo (MC) simulations.

The rest of this paper is organized as follows: Section 2 presents the related works in terms of the comparative SRAM cells. Section 3 explains the materials and methods used in the design of the proposed cell structure, as well as the working principles. Section 4 presents the various analyses, observations, and results of the proposed E^2^VR11T cell and comparative C6T [20], S8T [21], ST9T [22], LP10T [23], and MET11T [24] cells, with respect to dynamic power, leakage power, current and energy consumption, stability, delay time, area, PVT variations, and variability investigations through Monte Carlo simulation results and the Electrical Quality Metric (EQM). Finally, Section 5 concludes the paper.

## 2. Related Works

Traditional cells, such as conventional C6T and standard S8T cells, are industry-standard architectures [20,21] that are normally used to benchmark the performance of SRAM memory circuits. However, these cells suffer from conflict between write and read operations, stability degradation, write failures, half select issues, etc. Moreover, if the read static noise margin is improved in terms of read stability, then it could affect the write operation. It has also been forecasted by researchers that process variation may limit the required minimum voltage for read and write operations [18,24]. Researchers have proposed several SRAM topologies with improved outcomes when compared to traditional topologies [11,12,13,14,15].

In this work, the conventional C6T [20], standard S8T [21], Schmitt trigger ST9T [22], low-power LP10T [23], and multi-bit-error-tolerant MET11T [24] have been selected for comparison and benchmarking. All of the cells are designed at the schematic and at the layout levels using 45 nm technology and simulated to compare the results against the proposed E^2^VR11T cell. The operational specification of the selected cells, including the proposed cell, is highlighted in Table 1. The ongoing research confirms that the SRAM memory design and development is still progressing despite the many different cells with various applied techniques. Characteristics such as energy efficiency and process tolerance are very important for SRAM memory in terms of low-power applications.

The read and write operations of the selected cells are performed utilizing either a single-end decoupled read mode or differential write mode. There are two to three bit lines used in these cells. The word line (WL) and read word line (RWL) are commonly applied to enable write and read operations. There are two transistors used in the read path of the S8T [21] and MET11T [24] cells. Considering the highlighted challenges of existing cells, energy-efficient and variability-resilient 11T (E^2^VR11T) SRAM memory is proposed in this research work. The comprehensive process variation analysis of the proposed E^2^VR11T cell and the selected cells are discussed in detail, along with the analyses of energy consumption in this research. The variability investigation of all the cells against the proposed cell is carried out to prove the resilience of the cell. In addition to the comprehensive analysis of the proposed E^2^VR11T SRAM, several comparative analyses are also carried out with selected conventional C6T [20], standard S8T [21], Schmitt trigger ST9T [22], low-power LP10T [23] and multi-bit-error-tolerant MET11T [24] cells.

## 3. Materials and Methods

This research work proposes a novel design of an energy-efficient and variability-resilient SRAM (E^2^VR11T) cell. The proposed cell has been tested in schematics and implemented in layout together with other selected cells. A 4 × 4 memory array was designed together with peripheral circuits and implemented in the layout. The memory array functions according to the design and development of the technique. All of the selected memory cells and proposed SRAM were simulated in a similar environment for read, write, and hold operations. The results were comprehensively analyzed and discussed in terms of power, current, delay time, and stability. The process, voltage, and temperature variations were extensively examined to understand the tolerance of the proposed cell. Further, the energy consumption of the proposed cell was computed and verified to understand the energy efficiency of the cell.

**Figure 1:**
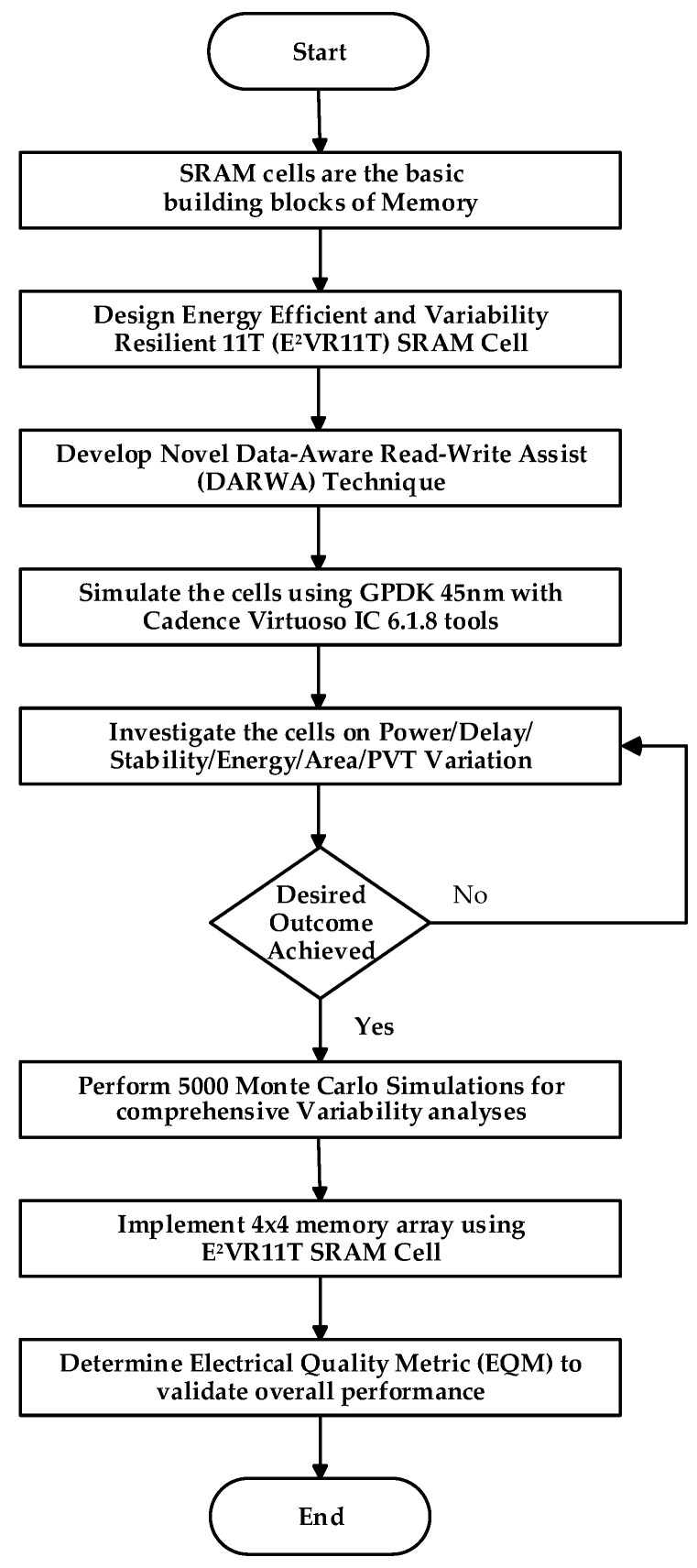
Flowchart of the methodology of the design of E^2^VR11T SRAM cell and the implementation of memory array.

Monte Carlo analyses were carried out using 5000 samples for all cells to understand the behavior and variability. From the mean and standard deviation results, variability is determined for read/write power, leakage power, leakage current, read/write current, and read/write delay time. The variability comparison was widely investigated to validate the resilience and robustness of the proposed SRAM. Next, the layout area was analyzed and presented. Finally, the electrical quality metric was found to confirm the overall performance of the proposed SRAM cell. Figure 1 shows the flow chart of the research methodology.

### 3.1. Data-Aware Read–Write Assist (DARWA) Technique

The novel Data-Aware Read–Write Assist (DARWA) technique was developed to reduce the power consumption of the cell and improve stability. This technique adopts two different operations, namely single-ended read operation and dynamic differential write operation; thus, the read and write operations can be performed by two isolated circuits. The isolation of two operations completely avoids read–write conflict.

- Single-Ended Read Operation

**Figure 2:**
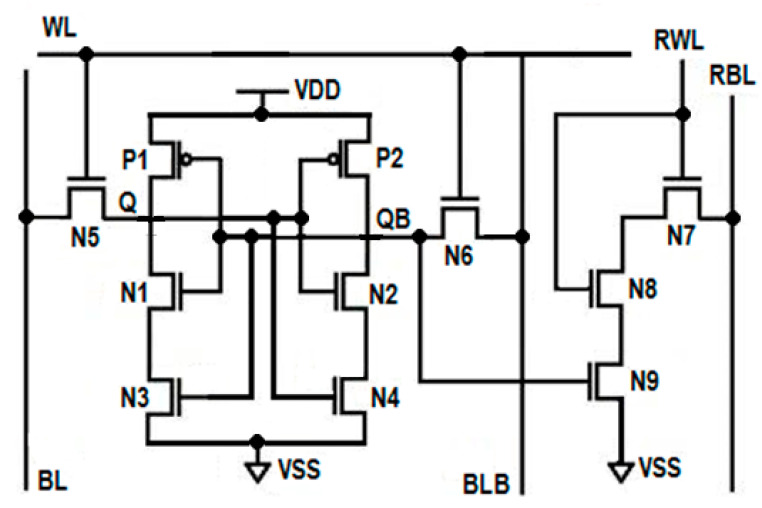
Schematic diagram of proposed E^2^VR11T Cell.

As shown in Figure 2, the single-ended read operation is performed by the independent read circuit, which is designed with three series connected NMOS transistors N7, N8, and N9. These three series connected transistors are further connected to the read bit line (RBL). The gate of transistor N9 is controlled by storage node QB, whereas the switching behaviors of transistors N7 and N8 are controlled by the read word line (RWL). During the write operation, RWL = 0, which disconnects the read circuit from the write circuit due to the OFF transistors N7 and N8, improves read stability. When QB = 0 and RWL = 0, all of these three series connected transistors turn OFF and offer very high resistance from RBL to the ground; thus, the leakage current is almost zero. This stack effect effectively controls the power consumption during the write operation. The isolation of the read circuit from the write bit lines enhances the read static noise margin (RSNM). During read 0 operation, due to OFF transistor N9, the current from RBL to the ground is limited, which reduces read dynamic power consumption.

- Dynamic Differential Write Operation

In the write circuit, there are two cross-coupled latches, namely the left side latch and the right side latch. The left side latch is formed by three transistors, P1, N1, and N3, whereas the right side latch constitutes transistors P2, N2, and N4, as shown in Figure 2. The gate of transistors P1 and N1 are connected and further connected to the common drain of transistors P2 and N2. This node is known as the write storage node, QB, whereas the common gate connection of transistors P2 and N2 is connected to the common drain connection of transistors P1 and N1. This node is known as the write storage node Q. Q and QB are complementary to each other. The storage nodes Q and QB are connected to the write bit lines BL and BLB through two access transistors, N5 and N6. The switching behavior of these two access transistors, N5 and N6, are controlled by the write word line WL. Transistors N3 and N4 are known as tail transistors, whose gates are connected to QB and Q, respectively. This arrangement forms the differential circuit. The introduction of two-tail transistors in the proposed cell makes the write circuit dynamic. Therefore, the write operation is performed dynamically with the differential circuit. During the write operation, depending upon the data at the storage nodes Q and QB, one of the tail transistors will turn OFF, which performs the write operation without waiting for BL or BLB to discharge completely and saves dynamic power considerably. The tail transistors in the write circuit enable to pull the logic either strong high or strong low at the storage nodes, which enhances the write ability of the cell. The induced stacking effect due to tail transistors on both sides of the inverters drastically minimizes the leakage power.

This technique is adopted to minimize the power consumption and leakage current and enhance the read stability and write ability of the cell. Further, the DARWA technique is proven to attain energy efficiency and variability resilience against PVT (process, voltage, and temperature) variation, power, and performance, and in return, achieves higher immunity, operational reliability, and overall performance.

### 3.2. Design of Energy-Efficient and Variability-Resilient 11T (E^2^VR11T) SRAM Cell

The proposed energy-efficient and variability-resilient SRAM (E^2^VR11T) cell, as shown in Figure 2, consists of eleven transistors (11T) and is designed with independent read and write circuits. The left side latch is formed by the P1 and N1 transistors, whereas the P2 and N2 transistors and their switching activity is controlled by the word line (WL). The main function of these two access transistors is to connect the bit lines BL and BLB to storage nodes Q and QB. There are two tail transistors N3 and N4 connected in series with pull-down transistors N1 and N2. The N3 and N4 transistors’ gate is connected to the output nodes QB and Q, respectively, to pull the logic either at a strong high or a strong low at the storage nodes, which enhances the write ability of the cell. In the write circuit, lower discharging activity at the respective bit line saves a considerable amount of dynamic power. During write 1, transistor N3 turns OFF and disconnects the path from V_DD_ to the ground, and hence no current will flow in this path. This OFF transistor N3 flips the voltage at Q to high without waiting for BL to discharge completely. Similarly, during the write 0 operation, OFF transistor N4 flips the storage node QB to high without waiting for BLB to fully discharge. The lower discharging activity at the respective bit line will save considerable dynamic power. The isolation of read and write circuits enhances the write ability. The OFF transistor also restricts the leakage current in the respective write path.

The single-ended read circuit is designed with three NMOS transistors, namely N7, N8, and N9, to improve the cell’s read stability. The transistors N7 and N8 behave as read pass transistors, and their switching behavior is controlled by RWL. During the read operation, RBL is pre-charged to V_DD_. For read 0 operation, all three series connected transistors turn ON, and hence RBL will discharge faster. Using a single-ended sense amplifier, the voltage drop at the RBL will be measured and interpreted as logic 0. The three series connected ON transistors make the cell read 0 faster. During read 1 operation, transistor N9 turns off, which disconnects the RBL from the ground and offers a very high resistive path that does not allow RBL to discharge. The absence of discharging activity at RBL is interpreted as logic 1, which saves dynamic power. Since the read and write operations are performed by different circuits, the read static noise margin (SNM) of the cell is improved drastically. The use of three transistors in the read path introduces the stack effect, which helps to reduce the leakage current in hold mode (RBL = V_DD_, RWL = 0 = WL, BL = V_DD_ = BLB). The current flow is also limited from the read bit line to the ground, and hence the read power dissipation is less. The status of control signals for the write, read, and hold state is presented in Table 2.

### 3.3. Working Principle of E^2^VR11T SRAM Cell

The proposed E^2^VR11T cell was designed and implemented into 45 nm complementary metal oxide semiconductor (CMOS) technology. The device size has been evenly applied to NMOS and PMOS transistors for a fair and reasonable comparison. Device sizes of 120 nm/45 nm and 150 nm/45 nm have been used for all the NMOS and PMOS transistors, respectively, for the proposed and all the comparative SRAM cells as well. This combination of device sizing provides the nominal voltage transfer characteristics (VTC). The supply voltage of 1 V at 27 °C temperature is applied for all the operations.

#### 3.3.1. Read Operation

The word line (WL) is not used in this read operation; hence, it is kept low. The bit lines, BL, BLB, and RBL, are pre-charged during the read operation, and RWL is connected to V_DD_. The single-ended read circuit would either perform read ‘1’ or read ‘0’ operation depending upon the output in node QB. The read circuit is also connected to the single-ended sense amplifier to interpret the data as logic 0 or logic 1.

**Figure 3:**
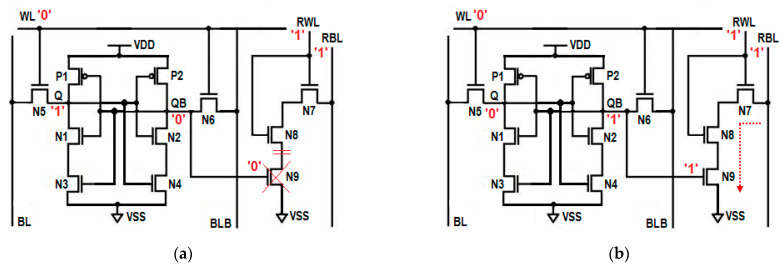
Read circuit; (**a**) read ‘1’ operation, (**b**) read ‘0’ operation.

When QB is holding output ‘0’, the NMOS transistors N7 and N8 in the read path are turned ON, whereas the last N9 transistor is OFF, which disconnects the RBL from the ground. The open read path presently does not allow the read bit line (RBL) to discharge; hence, it maintains its pre-charged voltage of 1 V, which is equivalent to read 1 operation. This voltage level is interpreted as logic 1 by a sense amplifier, which is equivalent to the read 1 operation. The absence of discharging activity on the RBL saves the dynamic power consumption during the read 1 operation. The OFF transistor in the read 1 path also restricts the leakage current and reduces static power consumption as well. The resulting circuit for the read 1 operation is shown in Figure 3a.

**Figure 4:**
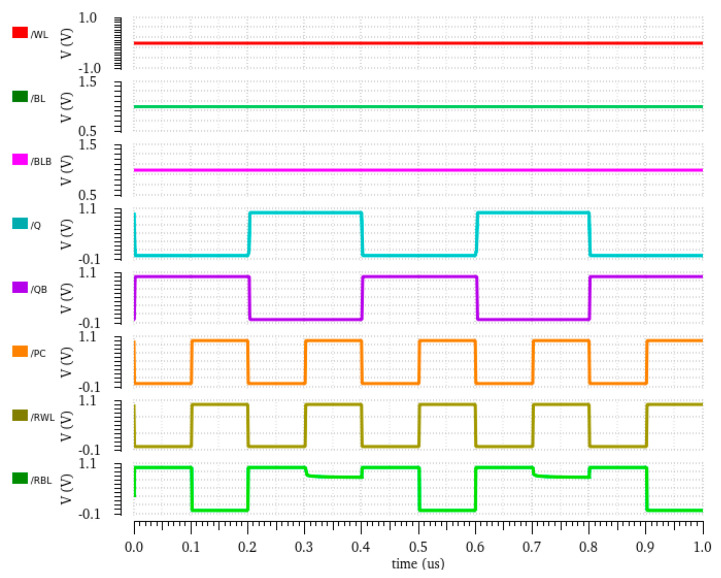
Read waveform of proposed E^2^VR11T cell.

When QB is stored with ‘1’, and when RWL is asserted, all three transistors in the read path are turned ON, which enables the full discharge of RBL, and the sense amplifier will now interpret this voltage fall on RBL, such as with read 0. The three series connected ON transistors offer a low resistive read path, which makes the reading of 0 faster compared to a conventional 6T SRAM cell and others. The resulting circuit for the read 1 operation is shown in Figure 3b. The transient response of the read operation is plotted in Figure 4.

#### 3.3.2. Write Operation

The world line WL is set to high, PC is kept low (to pre-charge the RBL), and the read word line RWL is also kept low once the input data are applied to bit lines BL and BLB. In the dynamic differential write operation, two tail transistors, N3 and N4, are included to pull the logic either on strong high or strong low at the storage nodes, which significantly enhances the write ability of the cell. The switching activity of these two tail transistors is controlled by logic at the storage nodes. The write noise margin (WNM) of the cell is found to be reasonably high, although the size of access transistors N5 and N6 did not vary.

**Figure 5:**
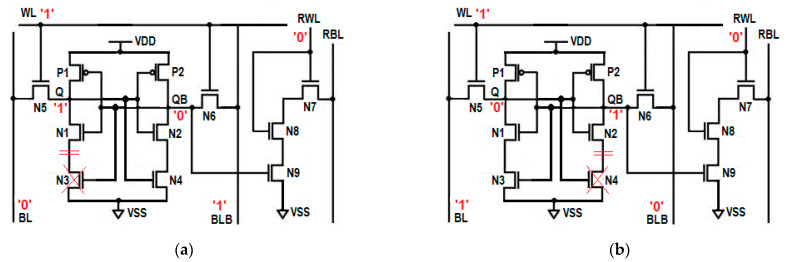
Write circuit; (**a**) write ‘1’ operation; (**b**) write ‘0’ operation.

To perform the write ‘1’ operation, the bit line of BL is kept low, BLB is kept high, and the word line WL is set to high. The ON access transistor N5 passes the logic low at the input of the left inverter P1 and N1, which yields high logic at Q and low logic at QB. A lower value at QB turns OFF the transistor N3, which offers a high resistive path and hence no current flows through this path and flips the storage node Q at a strong high without discharging BLB. The resulting circuit for write ‘1’ operation is shown in Figure 5a. The OFF transistor also restricts the leakage current in the path and saves static power as well.

**Figure 6:**
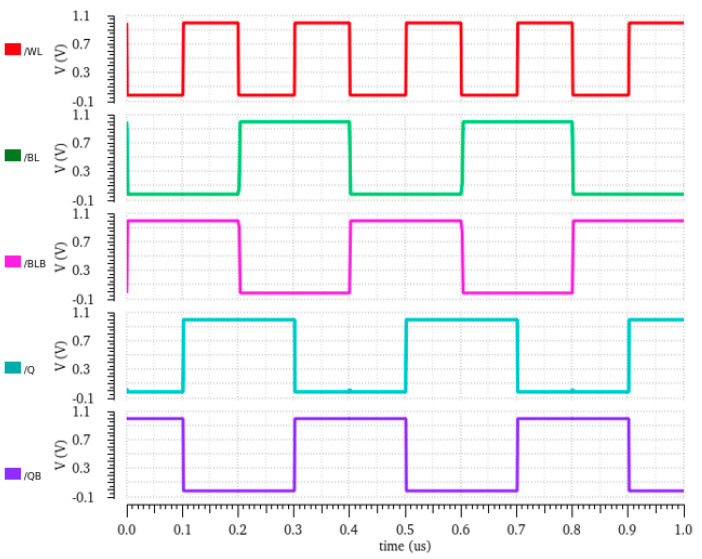
Write waveform of proposed E^2^VR11T cell.

For the write ‘0’ operation, the bit lines BL is set to high, and BLB is kept low. The high voltage at BL will flip the data at Q to low due to the high input at the left side inverter. The lower logic at storage node Q will turn OFF the tail transistor N4. The off transistor N4 offers a high resistive path, which flips the storage node QB high without allowing BL to discharge completely, which results in the low logic at Q, which is equivalent to the write ‘0’ operation. Figure 5b shows the equivalent circuit for performing write ‘0’ operation. Thus, the lower discharging voltage at the BLB saves considerable dynamic power. The transient response and status of bit lines during the write operation are plotted in Figure 6.

#### 3.3.3. Hold Operation

**Figure 7:**
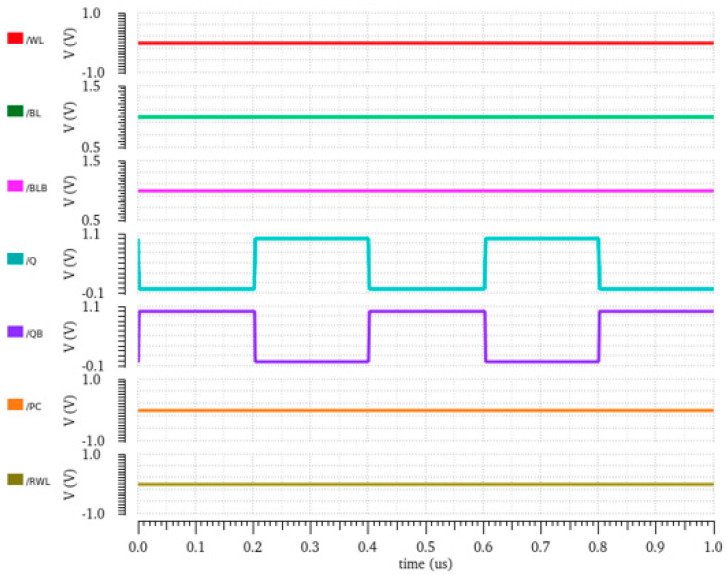
Hold waveform of proposed E^2^VR11T cell.

In the hold mode, both BL and BLB are pre-charged to V_DD_. The access transistors N5 and N6 are disconnected from the bit lines BL and BLB by setting WL to low. The RWL is also set to ground. Therefore, the cell is on standby or hold mode and will continue to hold the previously stored data at Q and QB. The transient response and status of outputs during the hold operation are depicted in Figure 7. The transistor sizing is maintained at a standard aspect and pull-up ratio. The aspect ratio and pull-up ratio have been verified for NMOS access transistors and for PMOS transistors against voltage transfer characteristics. The transistor sizing details are discussed in the next section.

## 4. Results and Discussions

### 4.1. Simulation Environment

Simulations of the proposed E^2^VR11T cell and comparative cells have been carried out using 45 nm CMOS technology with the Cadence virtuoso simulator tool. A 45 nm Generic Process Design Kit (GPDK) was used to perform various simulations to determine power consumption, access delay, process variation tolerance, performance on stability, area, and operating margin of all the cells. The temperature has been maintained at 27 °C with a 1.0 V supply voltage. Table 3 shows the various simulation parameters used.

The device size of PMOS and NMOS transistors is evenly applied for fair and rational comparison [25]. Energy efficiency has been analyzed to determine cell performance. PVT variation analyses are also performed in all process corners, from −50 °C to 150 °C temperature and a supply voltage from 500 mV to 1.0 V. Monte Carlo (MC) simulation has also been performed with 5000 samples to investigate the variability and impact of variability resilience of the proposed cell against other selected cells. The 10% variation of Gaussian distribution with 3σ is assumed in MC analysis.

### 4.2. PVT Variation Analysis

PVT (process, voltage, temperature) variation highly influences the behavior of SRAM cells in terms of power, switching speed, stability, and performance [26,27]. The transistor’s propagation time is determined by simulating PVT variation. A comprehensive analysis of PVT variations is carried out for all of the operations of the proposed and other comparative cells.

- Process Variation

The process variation is investigated at all process corners, namely nominal or typical–typical (TT), slow–slow (SS) corner, slow–fast (SF) corner, fast–slow (FS) corner and fast–fast (FF) corner for read, write operations, and compared with other considered cells. The process corner simulations are useful for identifying the parametric variation at the extreme level [28].

- Voltage Variation

By varying the voltage supply from 0.5 V to 1 V, the performance of the cell is analyzed to understand the behavior of the cell during the read, write, and hold mode. The inter-die variations would normally influence the change of threshold voltage. This may affect the performance of power, delay time, and stability. All of the cells are simulated with varying voltages for read, write, and hold operations. The energy consumption is also analyzed using different voltage levels to determine the actual impact of voltage variation on energy efficiency.

- Temperature Variation

The temperature variation from −50 °C to 150 °C is applied to identify the immunity level of the cells under different conditions and environmental settings. This is mainly undertaken to determine the suitability of the weather and adaptability to different environments. The variation in temperature is a crucial parameter to gauge the longevity of the cell and the endurance of its performance.

In this work, every parameter, such as read–write power, read–write current, leakage power, leakage current, and read–write delay time, was broadly investigated and analyzed in detail to determine the variability resilience. Further, PVT variation is also applied to the identified read–write energy and analyzed the energy efficiency of the proposed cell in detail to confirm the suitability of the proposed E^2^VR11T cell.

### 4.3. E^2^VR11T SRAM Power Consumption

Power consumption is a critical requirement for low-power applications as it executes many simultaneous applications that demand operational speed and overall system performance [29]. The power consumption of an SRAM device is approximately 80% of the system’s total power. The dynamic power and leakage power consumption of an SRAM and its connected devices are measured and analyzed to assess the overall memory performance. The dynamic power is highly influenced by the switching and supply voltage during the read–write operations due to the charging and discharging of capacitors. The dynamic power relationship can be defined by using [30]:

(1) $$P_{Dynamic}=C_{L}\times V_{DD}\times {∆V}_{BL}\times f$$

where *C_L_* is the lumped capacitance, *V_DD_* is the supply voltage, Δ*V_BL_* is the bit line voltage drop, and *f* is the frequency. The leakage or static power is measured during the hold mode when there is no switching due to the OFF transistors. The static power relationship is defined by the following [30]:

(2) $$P_{Leakage}=P_{Static}=V_{DD}\times I_{Leakage}$$

The power consumption analyses of the proposed cell and other comparative cells are discussed in the next sections.

#### 4.3.1. Dynamic Power Consumption

**Figure 8:**
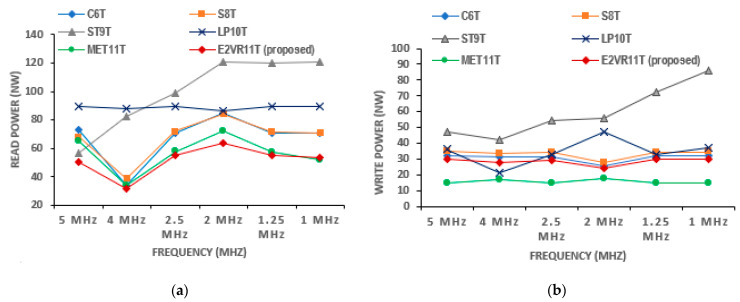
Dynamic power of cells versus frequency; (**a**) read power; (**b**) write power.

The power consumption of conventional C6T and standard 8T cells are quite high due to their bit line discharging activities. However, in the proposed E^2^VR11T SRAM cell, less power is consumed due to the lower discharging activity at bit lines. The dynamic power for both read and write operations are measured for proposed E^2^VR11T and other published cells for varying frequencies from 1 MHz to 5 MHz, as plotted in Figure 8. The proposed cell exhibits an average read power of 23.40%, 23.90%, 47.15%, 43.41%, and 8.84% lower than C6T, S8T, ST9T, LP10T and MET11T cells. The lower read power is due to lower discharging activity at RBL and the restricted leakage current. The average write power is also lowered by 7.80%, 14.68%, 67.28%, and 24.06% over C6T, S8T, ST9T, and LP10T cells. It can be observed from the graph that the proposed E^2^VR11T cell dissipates the lowest power for the read operation at all of the stipulated frequencies among the cells, and the write power is also lower, except for the MET11T cell when compared to the other cells.

#### 4.3.2. Read and Write Power Consumption

**Figure 9:**
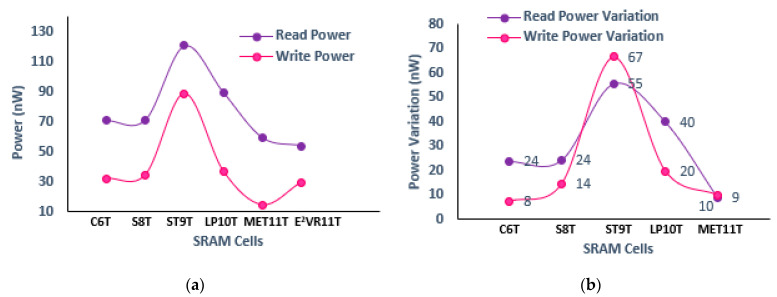
Power consumption of cells; (**a**) read and write power; (**b**) variation in read and write power against proposed E^2^VR11T cell.

The proposed E^2^VR11T cell dissipates less power due to lower discharging activity at the read and write bit lines. Both the read and write power of all of the cells is measured at 1 μs transient time and is plotted in Figure 9. It can be observed from the plot that the proposed cell dissipates the lowest read power among all of the cells. The lowest read power is caused by independent and single-ended read operation in the E^2^VR11T cell. The NMOS transistors, N7, N8, and N9, which are connected in series on the read path, perform quick read operation using RBL, which enables a power reduction. The read power is reduced by 23.87%, 24.19%, 55.42%, 39.94%, and 9.09% with a supply voltage of 1 V as compared to C6T, S8T, ST9T, LP10, and MET11T cells. This reduction is due to the stack effect as well as lower discharging voltage at the RBL, which reduces both static power as well as dynamic power.

From the power variation plot, it can be observed that write power is reduced by 7.52%, 14.37%, 66.60%, and 19.82% compared to C6T, S8T, ST9T, and LP10 cells due to the differential nature of the write circuit. The isolation of the write circuit from the read circuit restricts the overall leakage current. Lowering the leakage current reduces the overall static power consumption in the cell. The dynamic power of the cell is reduced due to the lower voltage drop at the respective bit line. The disconnecting effect of the feedback path of inverters and the shared column word line (CWL) and write word line (WWL) control signals are responsible for the lowest write power consumption of the MET11T cell.

#### 4.3.3. PVT Variation of Read and Write Power Analysis

**Figure 10:**
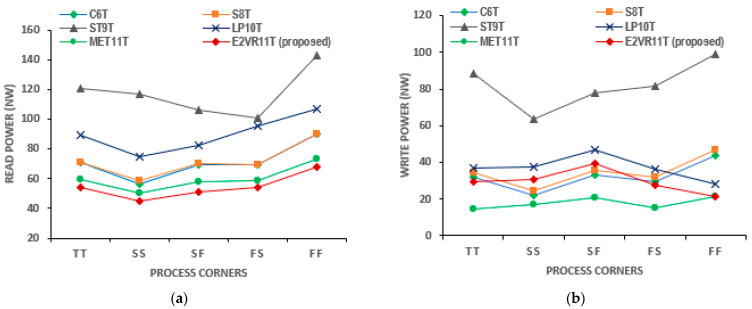
Process variation on power consumption of cells; (**a**) read power; (**b**) write power.

**Figure 11:**
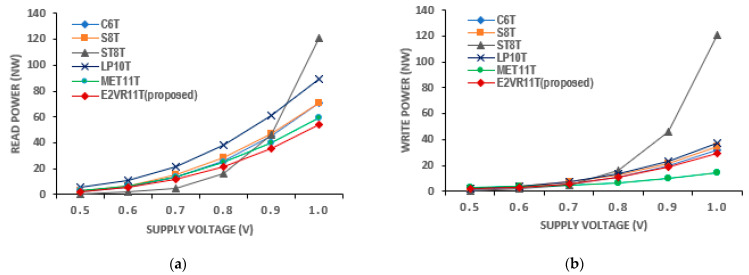
Voltage variation on power consumption of cells; (**a**) read power; (**b**) write power.

**Figure 12:**
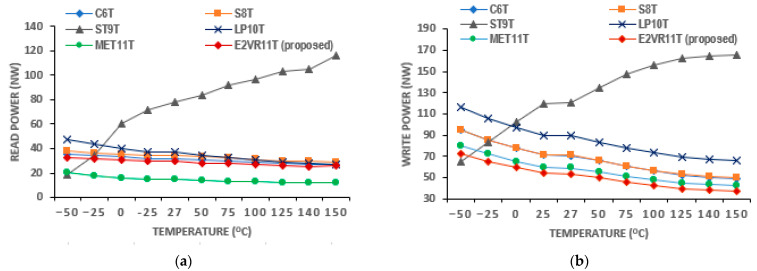
Temperature variation on power consumption of cells; (**a**) read power; (**b**) write power.

The read and write power simulation results taken from all process corners, voltage variations, and temperature variations are plotted in Figure 10, Figure 11 and Figure 12. The threshold voltage always changes due to the inter-die variations; hence, the power, performance, and stability are normally affected [31,32]. It can be observed from the plot that the proposed cell has the lowest read power in all of the process corners. The cell performs better in all the corners compared to other cells and improves the read stability and write ability. The average read power, 54.33 nW, of the proposed cell is again lower than all other cells in all of the process corners. The write power of the proposed cell is extremely low in the FF corner, which confirms that the cell is stable under the worst conditions as well. The MET11T has the lowest write power in all process corners.

The plot of the voltage variation shows that the read power of the proposed E^2^VR11T cell is the lowest at voltages from 0.5 V to 1.0 V. The write power is also lower next to the MET11T cell. The power increases as the supply voltage increases. The average read power dissipation of the proposed cell is lower by 31.19% and 42.11% against the ST9T and LP10T cells. The average write power of the E^2^VR11T cell is again lower by 13.89%, 63.50%, and 19.96% over the S8T, ST9T, and LP10T cells.

The plot of various temperatures ranging from −50 °C to 150 °C is shown in Figure 12. The proposed cell demonstrates the least write power at all temperatures and the second lowest for read operation after the MET11T cell. There is an improvement of 7.26%, 14.12%, 72.11%, and 18.77% as compared to the proposed cell’s 28.52 nW power for the write operation. The average read power of the proposed cell at all temperatures is 50.97 nW and lower by 23.59%, 24.10%, 60.56%, 40.31%, and 9.92% over C6T, S8T, ST9T, LP10T, and MET11T cells, respectively. This lesser sensitivity of the power against temperature in the proposed cell is due to the isolated read circuit that controls the leakage current effectively. From these results, it can be concluded that the proposed E^2^VR11T cell is PVT-compliant in terms of power consumption.

#### 4.3.4. Leakage Power Consumption

The leakage power consumption is normally quite a large percentage of the total power of the SRAM circuits as, most of the time, the cell remains in standby mode. It is a critical challenge due to the increase in leakage current on the transition points. The bit lines BL and BLB are charged to V_DD_, and the access transistors N5 and N6 are completely disconnected from the inverters. The word line WL and read word line RWL are kept low when the leakage power is measured. The leakage power of the E^2^VR11T cell is 52.2160 nW is the lowest among all of the selected cells. The induced stacking effect by N3 and N4 tail transistors on both sides of the latch minimizes the leakage power of the cell according to the DARWA technique. The leakage power of the proposed cell is reduced by 25.87%, 25.72%, 56.32%, 40.90%, and 9.71% compared to C6T, S8T, ST9T, LP10T, and MET11T cells, respectively, as shown in Table 4. The leakage power issue under the nanometer regime is a persistent challenge for memory designers. Therefore, this issue has been effectively handled in the proposed cell.

#### 4.3.5. PVT Variation of Leakage Power Analysis

**Figure 13:**
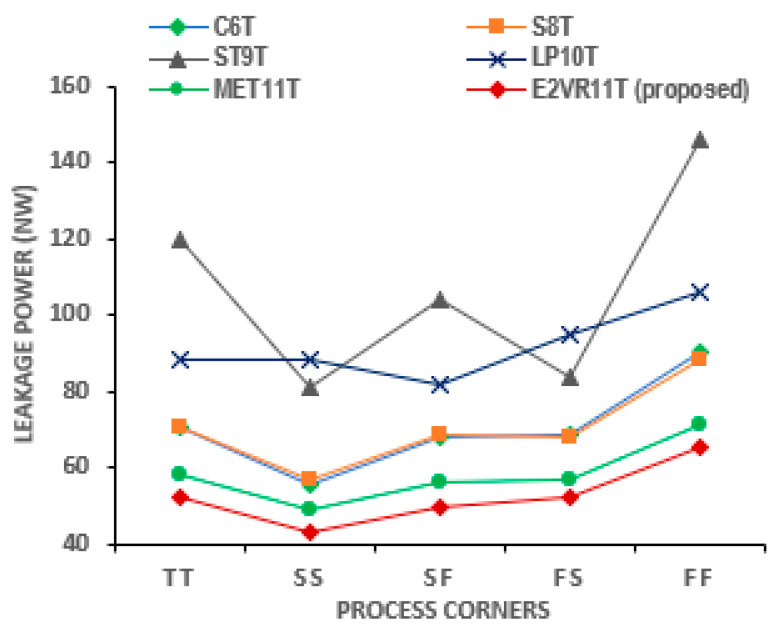
Process variation on leakage power consumption of cells.

**Figure 14:**
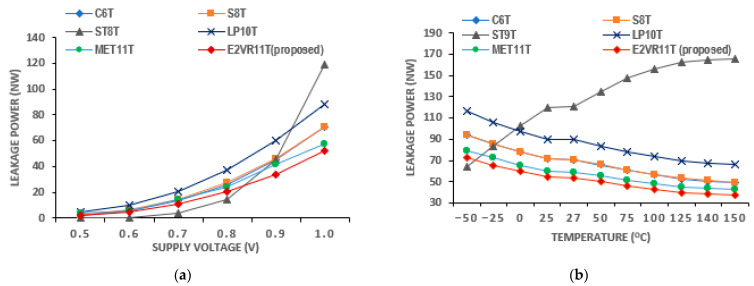
Leakage power consumption of cells; (**a**) voltage variation; (**b**) temperature variation.

The process variation simulation results of leakage power are plotted in Figure 13. The proposed cell’s average leakage power is the lowest at all process corners due to the stack effect induced by the tail transistors. The leakage power increases as the temperature increases [33]. The temperature variation would usually affect the performance and the speed of the cell [34]. The E^2^VR11T cell’s average leakage power of 52.52 nW is the lowest amongst all the cells at all corners, which can be seen from the plot. The voltage and temperature variations of the cells are plotted in Figure 14. The least leakage power in an average of 20.74 nW and 49.31 nW is demonstrated by the proposed cell at voltage variation and temperature variation. The effect of stacking transistors at the tail end of the write circuit and series connected NMOS transistors in the read circuit reduces the leakage power. This is a unique characteristic of the proposed SRAM cell in terms of the leakage power, as it is one of the major challenges of the SRAM design. The proposed E^2^VR11T SRAM highly demonstrates suitability in terms of leakage power under all PVT conditions and is immune to various environmental conditions with different voltages and temperatures.

### 4.4. E^2^VR11T SRAM Current

**Figure 15:**
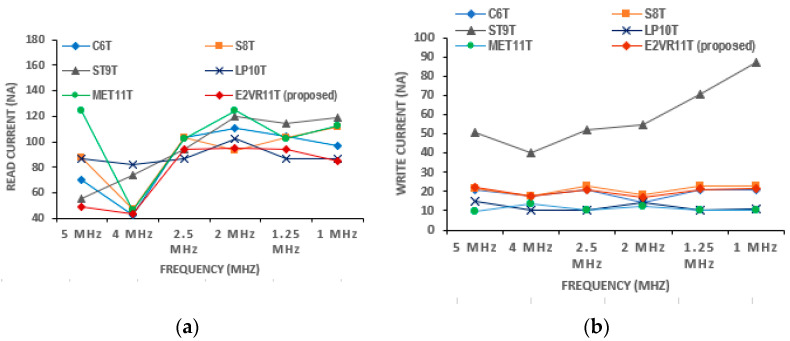
Cell current of various cells versus frequency; (**a**) read current; (**b**) write current.

The SRAM current is one of the significant design parameters. The proposed cell’s read and write currents have been investigated in detail and measured at 1μs transient time and are shown in Table 5. The current of all the cells at different frequencies from 5 MHz to 1 MHz is plotted for read and write operations in Figure 15.

It can be inferred from the table that the read current is lower compared to other cells. The average read current is lower by 13.01%, 29.48%, 20.39%, 27.04%, and 30.63% against C6T, S8T, ST9T, LP10T, and MET11T cells. On the other hand, the average write current is higher by 4.63% and 77.06% for S8T and ST9T cells. This enhancement is due to the isolated and single-ended read circuit.

#### PVT Variation of Current Analysis

**Figure 16:**
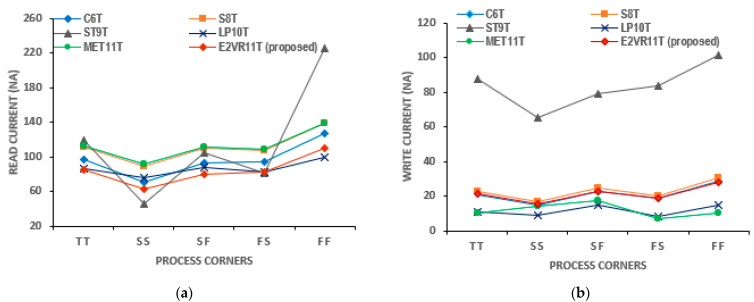
Process variation on current of cells; (**a**) read current; (**b**) write current.

The PVT variation in current is also applied and simulated for all of the cells. The currents on the process corners are plotted in Figure 16. It can be noticed that the maximum read current of 110.47 nA is achieved for the proposed cell in the FF corner, and the ST9T cell shows the highest read and write current at the FF corner out of all of the cells.

**Figure 17:**
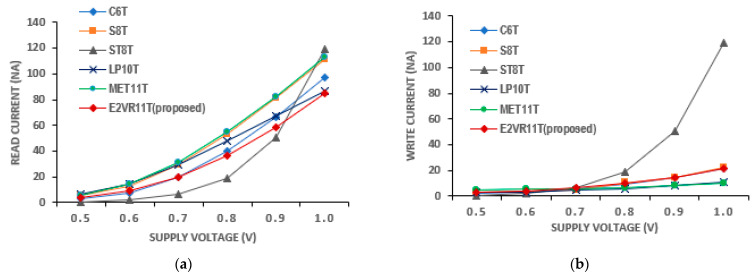
Voltage variation on current of cells; (**a**) read current; (**b**) write current.

**Figure 18:**
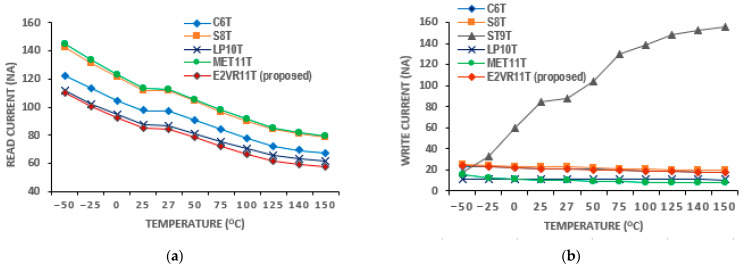
Temperature variation on current of cells; (**a**) read current; (**b**) write current.

The voltage variation in the read and write current is plotted in Figure 17. It is also observed from the voltage plot that the current gradually increases as the supply voltage increases. The lowest read current is achieved by the ST9T next to the C6T cell at 0.5 V. The proposed cell achieves a lower read current at 0.8 V, 0.9 V, and 1.0 V. The mean read current of 35.22 nA of the proposed cell is the lowest when compared to other cells, except for the ST9T cell. The mean write current of 9.75 nA is almost equal to C6T and S8T cells. The LP10T cell achieves the least mean write current of 5.99 nA at all supply voltages. The temperature variation results of the read and write current together with the other cells are plotted in Figure 18. On the temperature variation, the proposed cell proves to be immune to any condition as it achieves the minimum mean read current. The ST9T cell shows the highest current for the range of temperatures. However, the mean read current of 78.90 nA and the mean write current of 20.52 nA of the proposed E^2^VR11T cell shows that it can work at different temperatures without any disturbance.

### 4.5. E^2^VR11T SRAM Delay Time

**Figure 19:**
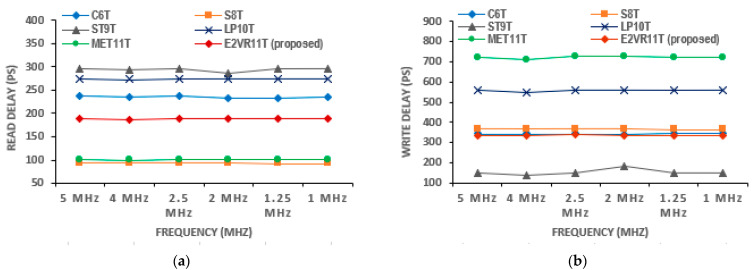
Delay time of various cells versus frequency; (**a**) read delay time; (**b**) write delay.

The speed and performance of the SRAM cell are normally dictated by delay time or access time. The read delay time (R_DT_) is calculated between RWL and RBL from the first rising or falling edge with a 50% threshold voltage. The write delay time (W_DT_) is measured between the WL and Q. The read and write delay time of the cells is measured from 5 MHz to 1 MHZ frequency and plotted in Figure 19. The mean read delay of the proposed cell is improved by 32.75%, 36.04%, 31.04%, and 31.46% at all frequencies compared to the S6T, ST9T, and LP10T cells. However, the S8T and MET11T cells show less read delay than the E^2^VR11T cell. The mean write delay time is also improved by 32.11% and 53.32% over the LP10T and MET11T cells, respectively.

#### 4.5.1. Read Delay Time (R_DT_)and Write Delay Time (W_DT_)

**Figure 20:**
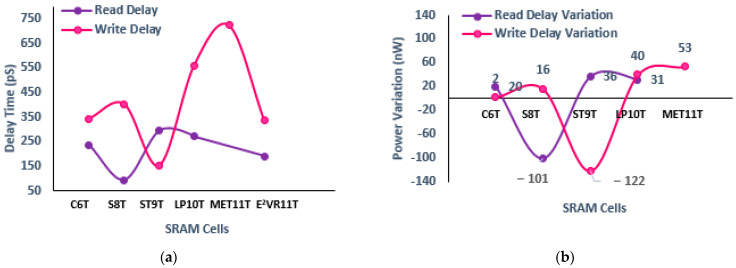
Delay time of cells; (**a**) read and write delay; (**b**) variation in read and write delay against proposed E^2^VR11T cell.

The read and write delay time measured at 1 μs transient time is plotted in Figure 20. It can be deduced from the plot that and read delay of the proposed cell has improved by 20.36%, 36.37%, and 31.35% against C6T, S8T, and LP10T cells due to the independent single-ended read circuit. S8T has recorded the lowest R_DT_ of 93.66 pS, and MET11T has the largest R_DT_ of 100.908 nS. The dynamic differential write operation of the proposed cell implies the improvement of write delay time (W_DT_) of 1.95%, 16.21%, 39.87%, and 53.36% over C6T, S8T, LP10T, and MET11T cells. The ST9T cell records the lowest W_DT_. The delay time variation against other cells is also presented for read and write operations.

#### 4.5.2. PVT Variation of Delay Time Analysis

**Figure 21:**
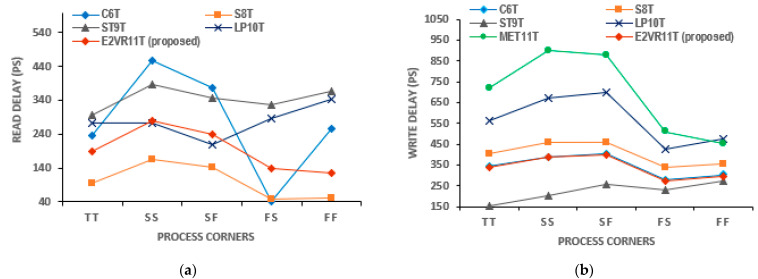
Process variation on delay time of cells; (**a**) read delay; (**b**) write delay.

The variation analyses are also performed for the delay time as it is critical for the speed and performance. The delay time findings on process corners have been plotted in Figure 21. It is observed that the proposed cell records the lowest R_DT_ and W_DT_ in the FF corner due to the DARWA technique. The C6T cell shows the lowest R_DT_ in the FS corner and the largest in the SS corner. Similarly, ST9T depicts the lowest W_DT_ in the TT corner, with MET11 being the highest. The R_DT_ and W_DT_ of the proposed E^2^VR11Tcell is reasonably low, and thus, the delay time has improved when compared to the other cells.

**Figure 22:**
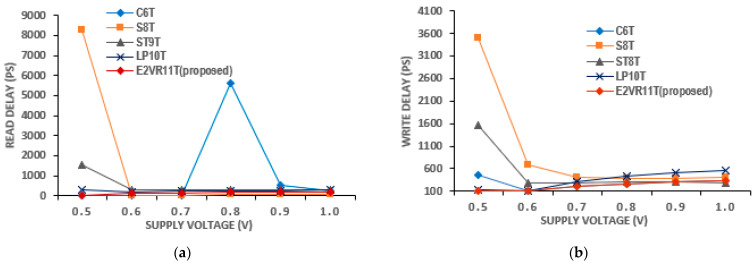
Voltage variation on delay time of cells; (**a**) read delay; (**b**) write delay.

**Figure 23:**
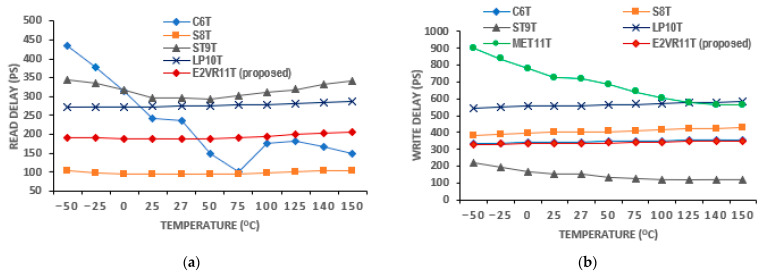
Temperature variation on delay time of cells; (**a**) read delay; (**b**) write delay.

Regarding voltage variation, the delay time does not reflect properly at a low voltage range. The findings related to varying voltage from 0.5 V to 1.0 V are plotted in Figure 22. As can be seen from the plot, the C6T and S8T show different ranges of R_DT_. However, the proposed cell works fine and shows the enhancement of R_DT,_ and it progresses as the supply voltage increases. The W_DT_ of S8T records the largest and the largest by MET11T. The proposed cell shows a consistent improvement in W_DT_. As all the other cells work in a differential write operation, they demonstrate a reasonably low W_DT_ as in 0.5 V and 1.0 V when compared to the E^2^VR11T cell. The delay time on the temperature variation is shown in Figure 23. The S8T cell shows the least R_DT_, and ST9T shows the largest R_DT_. The ST9T shows the least, and MET11T depicts the largest W_DT_. However, the proposed cell demonstrates a reasonably low W_DT_ and proves again the fitment of any environmental conditions with temperatures from −50 °C to 150 °C.

### 4.6. E^2^VR11T SRAM Energy Consumption

The SRAM memory’s energy efficiency is determined based on energy consumption. The energy consumption of SRAM is calculated based on the respective power and delay. The product of write power and write delay time is the write energy and is normally measured in the unit of joules [35]. Similarly, the read energy is determined based on the read power and read delay time. In this section, the energy has been analyzed in detail to understand the energy efficiency of the proposed and comparative cells. In addition, the PVT variation is also analyzed for all the cells to determine the cell’s robustness against energy.

#### 4.6.1. Read and Write Energy

**Figure 24:**
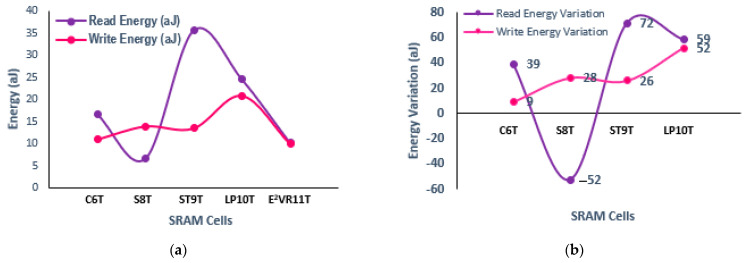
Energy consumption of cells; (**a**) read and write energy; (**b**) Variation on read and write energy against proposed E^2^VR11T cell.

The determined results of the read and write energy of all the cells are plotted in Figure 24. The proposed cell attains the read energy of 10.124 aJ and write energy of 9.974 aJ, which is comparatively lesser than other cells. The energy consumption and energy variation are plotted as well. It can be deduced from the plot that due to the single-ended read circuit and quicker discharge of RBL, the read energy of E^2^VR11T is 39%, 72%, and 59% lower than C6T, ST9T, and LP10T cells, respectively. The dynamic differential write operation with faster switching and no additional signals contributes to the lower write energy of the proposed cell. The write energy of E^2^VR11T is 9%, 28%, 26%, and 52% lower than the C6T, S8T, ST9T, and LP10T cells.

#### 4.6.2. PVT Variation of Energy Analysis

**Figure 25:**
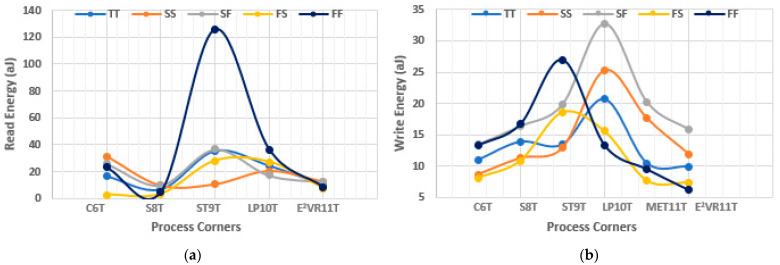
Process variation on energy consumption of cells; (**a**) read energy; (**b**) write energy.

The PVT variation is also applied to the energy to determine the energy efficiency and variability resilience of the cell with respect to process, voltage, and temperature. The process variation in energy is plotted in Figure 25. The proposed cell performs better in all the corners and records the least read energy. The ST9T records the highest read energy in the FF corner, and all the cells show an increase in write energy in the SF corner. Otherwise, all of the cells show a similar trend, such as read/write delay in different process corners, as the energy is the product of power and delay.

**Figure 26:**
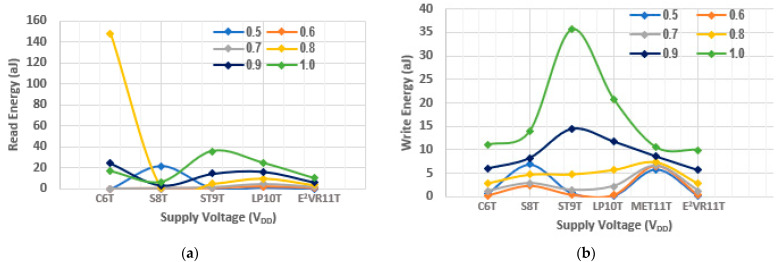
Voltage variation on energy consumption of cells; (**a**) read energy; (**b**) write energy.

The voltage variation in energy is plotted in Figure 26. The read and write energy at the lower supply voltage are not accurate due to either no delay or negligible delay time. However, it is stable as measured from 0.7 V for all of the cells. The V_DD_ variation depicts that the energy is in the range of 1 to 36 aJ, except for the C6T cell at 0.8 V. It shows the same trend of power and delay time at V_DD_ variation. The proposed cell again proves that it is stable and energy efficient during voltage variation.

**Figure 27:**
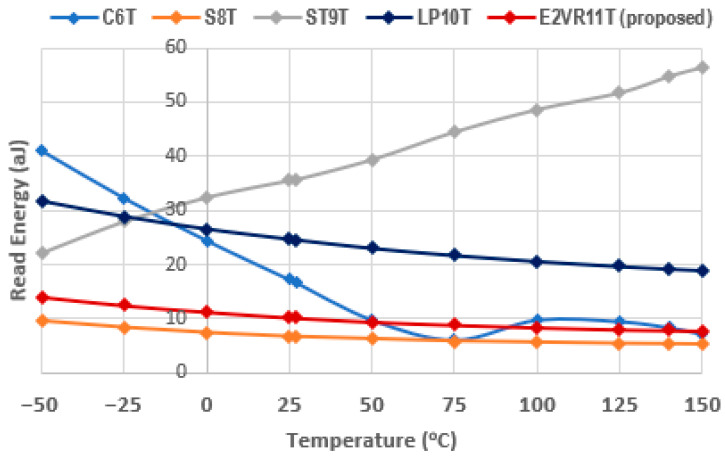
Temperature variation on read energy consumption of cells.

**Figure 28:**
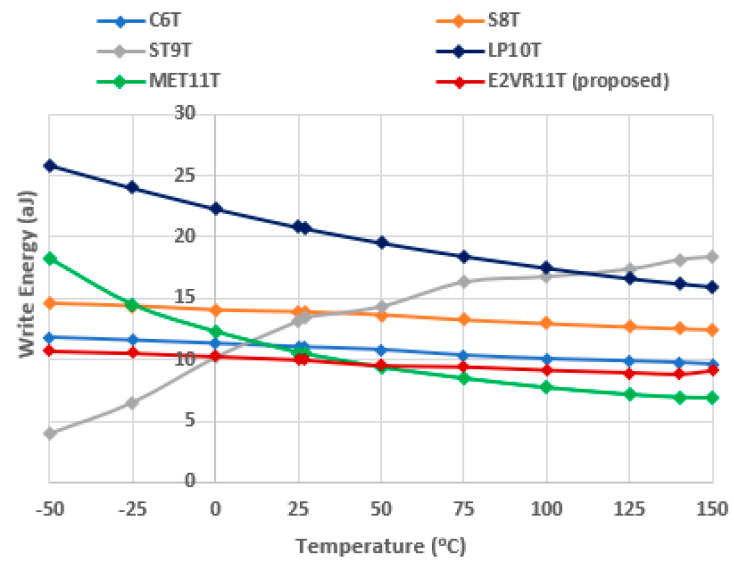
Temperature variation on write energy consumption of cells.

The computed result of the variation in temperature up to 150 °C for read energy is plotted in Figure 27, and for write energy, it is plotted in Figure 28. From the plot, it can be understood that the proposed cell has the least read energy of 7–14 aJ next to the S8T cell and the write energy of 9–11 aJ next to ST9T at the −50 °C extreme temperature. The proposed E^2^VR11T SRAM illustrates the strongest energy at all temperature ranges, which conforms the performance, speed, and efficiency towards the environmental immunity under different conditions.

### 4.7. E^2^VR11T SRAM Stability

Stability enhancement is one of the vital aspects of SRAM memory. There are three modes of SRAM stability depending upon the operating conditions. They are read, write, and hold modes, and their data margins are discussed through the Static Noise Margin (SNM). The SNM has been used to examine the stability of E^2^VR11T and selected cells in this section. The SNM is the amount of DC noise at the lowest level required to flip a stored bit in a node. During the hold, read, and write operations, the SNM is typically assessed and is referred to as Hold Static Noise margin (HSNM), Read Static Noise Margin (RSNM), and Write Noise Margin (WNM). It is noted that a greater SNM value for all operations can increase the robustness of the SRAM cell [36]. The static noise margin in this work is calculated using the conventional butterfly curve method [37].

#### 4.7.1. Read Stability

**Figure 29:**
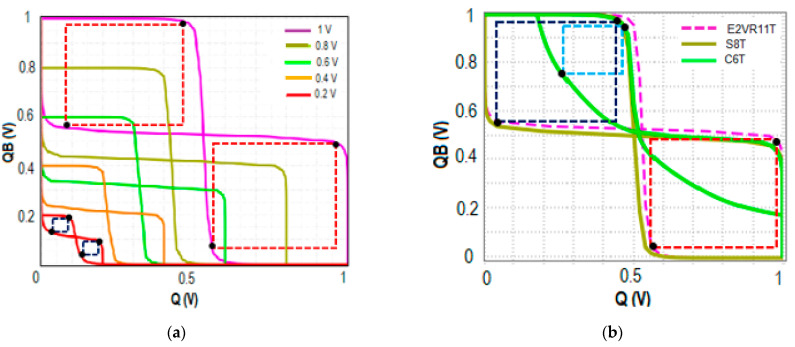
RSNM butterfly plot; (**a**) RSNM plot of the E^2^VR11T cell at V_DD_ variation; (**b**) RSNM plot of 6T, 8T and E^2^VR11T cells at 1 V.

The highest DC voltage that a cell may endure without losing the data during a read operation is defined as the Read Static Noise Margin. In this work, the RSNM is measured at 1 V when bit lines BL and BLB are connected to V_DD_ (1 V), the word line WL is connected to the ground (0 V), the read word line RWL is connected to V_DD_ (1 V), and RBL is pre-charged. The output nodes of Q and QB are not impacted by the single-ended read circuit with series connected transistors N7, N8, and N9, which achieved a higher RSNM. The RSNM of the proposed cell at V_DD_ variation from 0.2 V to 1.0 V is plotted to understand the stability of the proposed cell. Figure 29 shows the comparative RSNM butterfly curve of the proposed cell, C6T and S8T cells. The computed RSNM of the proposed E^2^VR11T cell is 470 mV and 400 mV for S8T and 160 mV for C6T cells. It is quite evident from the butterfly plot that the proposed cell achieves higher RSNM and an enhancement of 1.94x and 0.18x when compared to C6T and S8T cells. E^2^VR11T cell attains enhanced read stability due to the isolated read circuit and controlled leakage current. 

#### 4.7.2. Write Stability

**Figure 30:**
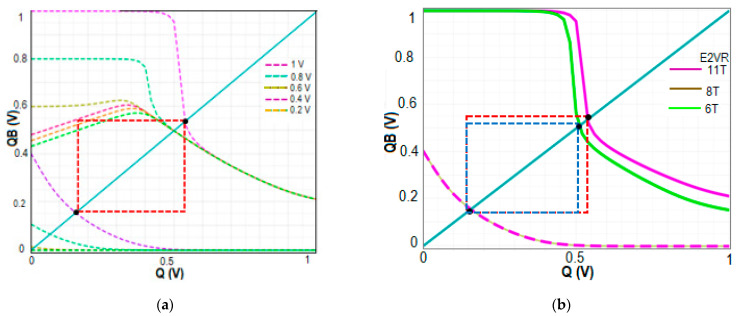
WNM butterfly plot; (**a**) WNM plot of the E^2^VR11T cell at V_DD_ variation; (**b**) WNM plot of 6T, 8T and E^2^VR11T cells at 1 V.

The write noise margin (WNM) is used to predict the SRAM cell’s write ability [38,39]. The bit lines of BL and BLB are alternately assigned with 0 and 1 for write 1 and 0 operations while WL is connected to V_DD_ (1 V) and the read word line RWL is connected to the ground. The WNM is calculated at 1 V supply voltage for write 1 and write 0 operations. The WNM of the proposed E^2^VR11T, C6T, and S8T cells are plotted in Figure 30 for V_DD_ variation to understand the write ability at different supply voltages. The proposed cell depicts a 460 mV WNM value compared to 420 mV and 370 mV for S8T and C6T cells. This improvement is due to the differential nature of the cell, isolation from the read circuit, and lower discharging activity at tits respective bit line. The reported achievement is 8.70% and 19.57% against S8T and C6T cells.

#### 4.7.3. Hold Static Noise Margin (HSNM)

**Figure 31:**
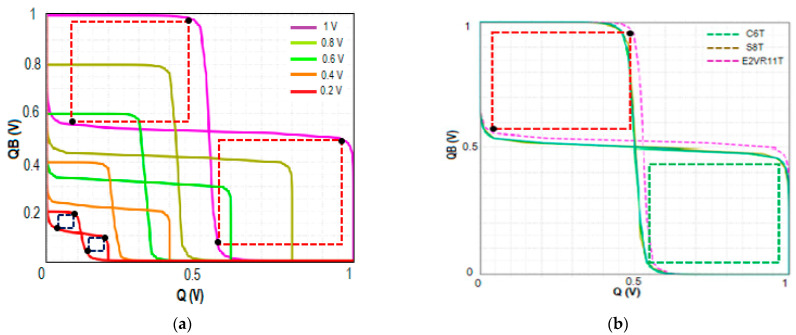
HSNM butterfly plot; (**a**) HSNM plot of the E^2^VR11T cell at V_DD_ variation; (**b**) HSNM plot of 6T, 8T and E^2^VR11T cells at 1 V.

The SRAM memory is normally in standby mode most of the time. Therefore, data retention is extremely important during the hold state. The stability at the hold mode is determined through HSNM. The minimum DC voltage that a cell can sustain without losing data in the hold mode is known as HSNM. It is normally measured during standby mode when both BL and BLB are connected to V_DD_ (1 V), the word line WL, and the read word line RWL are connected to the ground (0 V). The HSNM of the cells is plotted in Figure 31 for different V_DD_ to determine and understand the stability variation. It can be observed that HSNM of C6T and S8T cells are the same as 400 mV as it depends on the latching inverters. The proposed E^2^VR11T cell’s HSNM is 420 mV, which shows a 5% improvement over the other two cells due to the separate single-ended read circuit.

### 4.8. E^2^VR11T SRAM Variability Investigation

In addition to PVT variation analyses, the comprehensive study on the impact of variability analysis is highly important to determine the variability resilience of the SRAM. The variability can be utilized to understand cell behavior and to define the utility of bit cells in the nanometer regime. The variability investigation is significant for analyzing and understanding the performance of the cells. The suggested cell’s robustness, effectiveness against PVT variation, and resilience effect for read, write, and hold operations at a 1 V supply voltage and at a 27 °C temperature are assessed by performing Monte Carlo (MC) simulations. MC simulations are highly essential to estimating and evaluating SRAM performance accurately and efficiently under statistical variability. While running the MC simulation on 5000 samples for the read, write, and hold operations of all the cells, the Gaussian distribution with a 3σ variation of 10% is applied.

#### 4.8.1. Power Variability

Power variability is applied as a main parameter to evaluate the process tolerance and resilience of the cells [40,41]. The MC simulation on power variability was carried out with 5000 samples on random variation for read and write operations. The read and write power variability analyses and comparative results of all the cells, inclusive of mean (μ) and standard deviation (σ) with respect to process and mismatch variations, are presented in Table 6.

**Figure 32:**
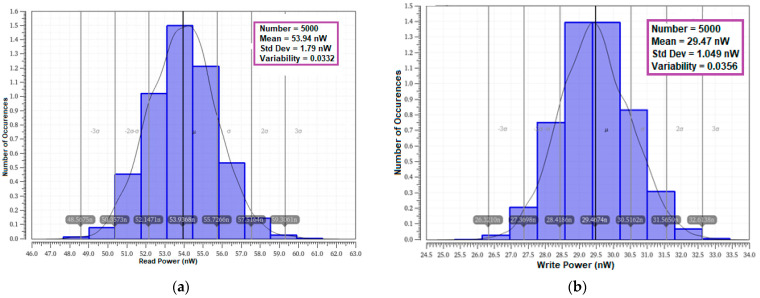
Monte Carlo simulation (5000 samples) of power variability plot of E^2^VR11T; (**a**) Read power variability; (**b**) write power variability.

The statistical variability analyses outcome and distribution for read and write power is plotted in Figure 32. It is noticed from the results that a larger value of the mean with lower value of standard variation is obtained for the proposed cell. The higher mean value normally reflects the robustness of the cell against the random variation. It can be inferred from the table that the mean read power of the proposed E^2^VR11T cell is 42% and 47% less and the mean read power is less by 24% over 6T/8T, 55%, 40%, and 9% against C6T, ST9T, LP10T, and MET11T cells. The variability (μ/σ) is calculated by dividing the mean by standard deviation to verify the resilience at random variation [42,43,44,45]. It is evident that the variability of the E^2^VR11T cell is 0.0332 for read and 0.0356 for write operations, which is reasonably lower than for other cells. Overall, the proposed cell’s lower variability compared to other cells at random variation confirms that it is highly reliable and robust for the appropriate applications.

#### 4.8.2. Leakage Power and Leakage Current Variability

**Figure 33:**
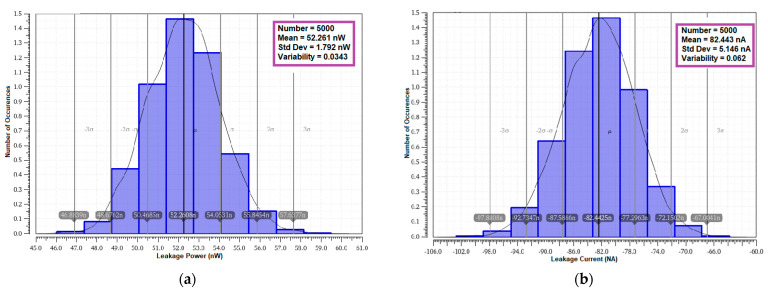
Monte Carlo simulation (5000 samples) variability plot of E^2^VR11T; (**a**) Leakage power variability; (**b**) Leakage current variability.

The variability in the leakage power and leakage current is performed with 5000 samples to determine the variation. The mean and standard deviation results from the MC analysis are presented in Table 7. The MC simulation outputs are plotted in Figure 33. It can be deduced from the table that the proposed cell exhibits the lowest mean leakage power and mean leakage current than other cells. The variability on leakage power is 0.0343 and 0.0624 for leakage current. The stacking effect causes minimized variability over other cells. The lower variability of the proposed cell addresses the bigger the challenge of leakage power reduction.

#### 4.8.3. Current Variability

**Figure 34:**
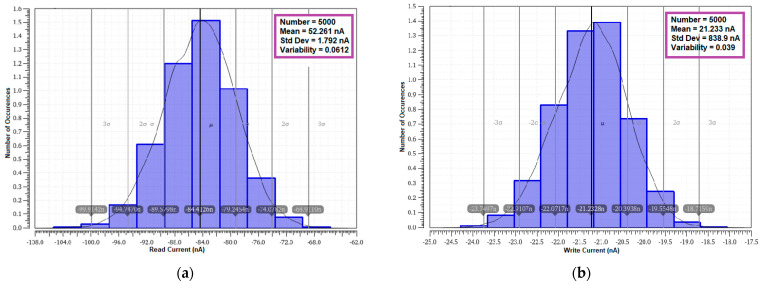
Monte Carlo simulation (5000 samples) of current variability plot of E^2^VR11T; (**a**) read current; (**b**) write current.

The variability analysis on the read and write current is also carried out with MC simulation on 5000 samples at random variation. The lesser mean read current is achieved by the proposed cell. The read and write current variability results are shown in Table 8, and the respective statistical distribution outcome is plotted in Figure 34. The MET11T cell depicts the lowest write current variability next to S8T. The write current variability of 0.0395 and read current variability of 0.0612 are achieved by the proposed cell due to limited leakage current and lower voltage drop at the bit line.

#### 4.8.4. Delay Time Variability

**Figure 35:**
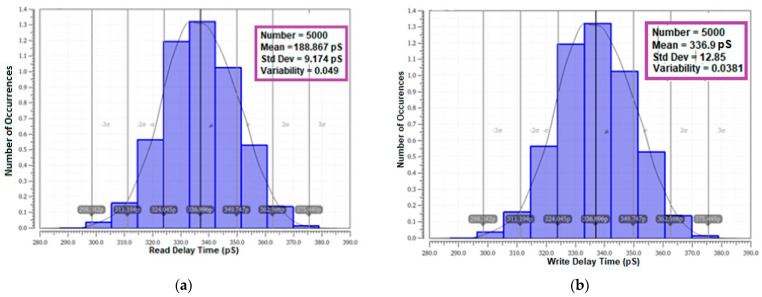
Monte Carlo simulation (5000 samples) delay time variability plot of E^2^VR11T; (**a**) read delay time; (**b**) write delay time.

The variability in the read and write delay time is another important parameter to be considered for investigation, as it relies on the speed and performance of the cell. The read and write delay times are also tested using MC simulation with 5000 samples. The results are presented in Table 9. and the histogram graphs are plotted in Figure 35. The variability results of the proposed cell 0.0486 and 0.0381 for read and write delay time affirm the enhanced speed and improved stability over other cells in the literature.

The E^2^VR11T circuit functions as per the design and technique and therefore it resulted with competent margins. The MC simulation of 5000 samples on statistical variability and its outcomes in terms of lower values demonstrate that the E^2^VR11T cell is highly variability resilient and tolerant to PVT variations. Overall, the variability investigative analyses on power, current, and delay time affirm that the proposed cell is stable and highly suitable for array design.

### 4.9. E^2^VR11T SRAM Layout Area Considerations

**Figure 36:**
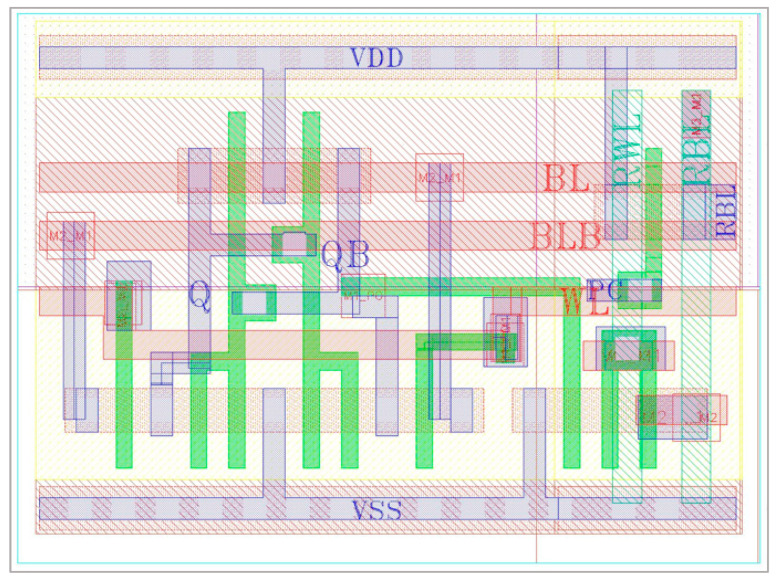
Layout of proposed E^2^VR11T cell.

**Figure 37:**
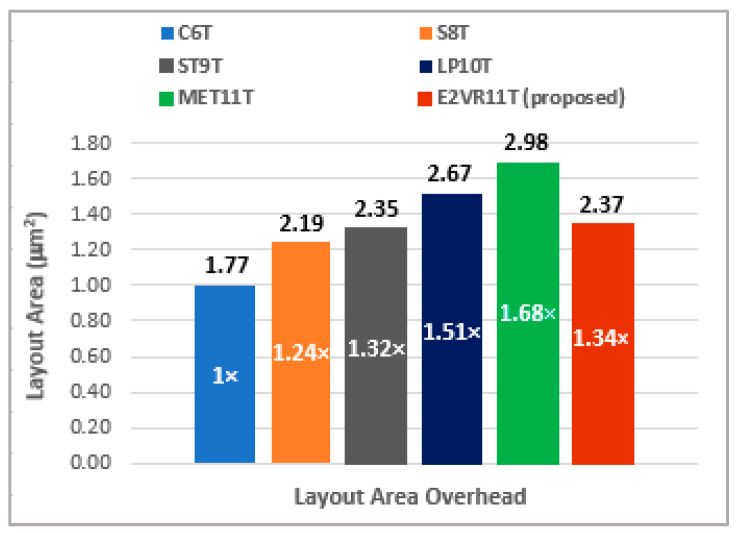
Layout area overhead of the cells against normalized conventional C6T cell.

The proposed E^2^VR11T cell layout is shown in Figure 36. All of the comparative cells and proposed cell layouts are constructed using 45 nm CMOS technology with an applicable design rule check (DRC). The connections on the layout versus schematic (LVS) have been verified and then parasitic extraction has been carried out. An area overhead of 1.34× is exhibited by the proposed E^2^VR11T cell as compared to the normalized area of conventional C6T cell due to an increased number of transistors. The other cells illustrate 1.24×, 1.32×, 1.51×, and 1.68× against the S8T, ST9T, LP10T, and MET11T cells, respectively. The DARWA technique approach of the proposed cell minimizes the area due to the independent read–write circuits arrangement. The minimum size transistors are used in the proposed cell with 11 transistors. The layout size is considerably less when compared to the MET11T cell, which also has a similar number of transistors. Figure 37 depicts the area overhead of all the cells against the C6T cell.

### 4.10. E^2^VR11T SRAM Memory Array

**Figure 38:**
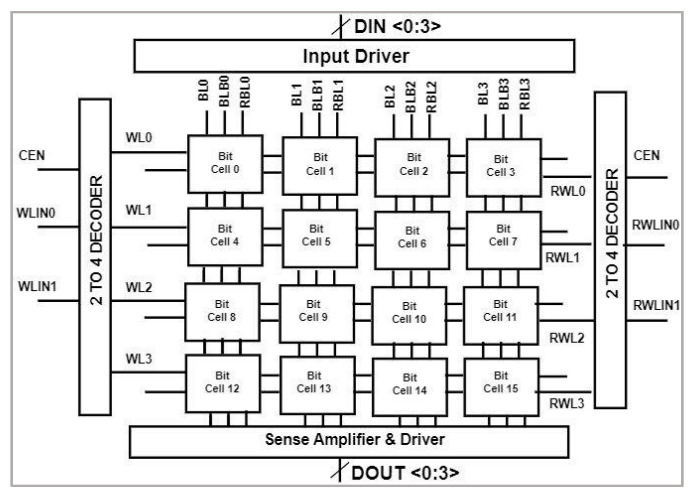
Block diagram of 4 × 4 memory array with peripheral circuits.

The proposed SRAM 4 × 4 memory array is implemented using the proposed cell in this section, as shown in Figure 38. The 2:4 decoder is used to select the row and column through the WL and RWL. The input/output drivers are used to drive the data and single-ended sense amplifier is used to interpret the data at the RBL during read operation. The array has been evaluated and verified for write and read operations.

**Figure 39:**
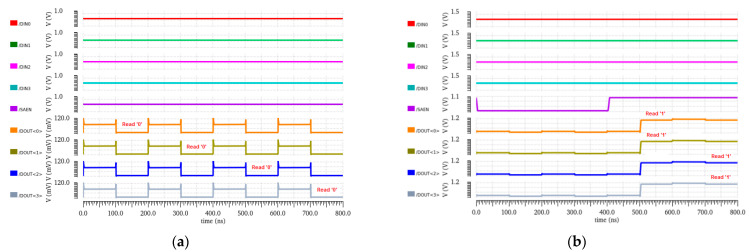
Transient response of 4 × 4 array; (**a**) Dataset 0000; (**b**) Dataset 1111.

The implemented array has been simulated for two read and write operations. The data inputs are set at the input nodes DIN0, DIN1, DIN2, and DIN3. These data bits are written in the selected row depending upon the decoder address. The same data stored in output Q nodes are readout through the sense amplifier. The sense amplifier interprets the output through DOUT0, DOUT1, DOUT2, and DOUT3. The total power consumption and current is presented in Table 10. The respective transient response at 800 ns is shown in Figure 39.

### 4.11. E^2^VR11T SRAM Electrical Quality Metric (EQM)

The Electrical Quality Metric (EQM) has been utilized for evaluating the overall performance of SRAM cells [46]. The EQM is calculated by using the following formula where:

(3) $$EQM=\frac{HSNM\times RSNM\times WNM}{Read\;Delay\times P_{Leak}\times P_{Read}\times P_{Write}\times Area}$$

**Figure 40:**
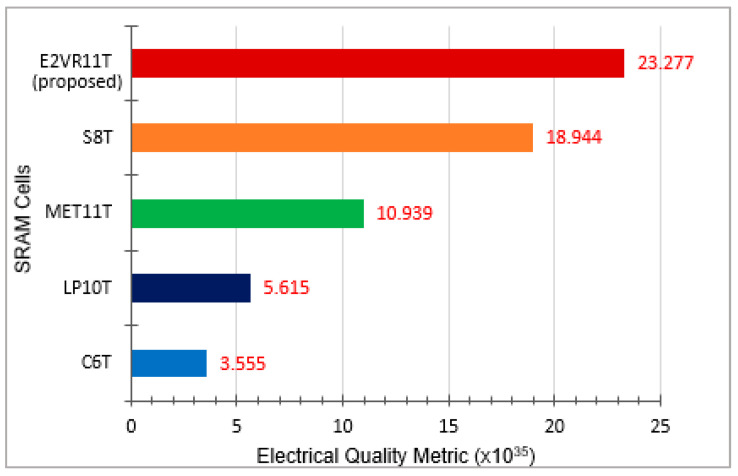
Electrical Quality Metric (EQM) of SRAM cells.

RSNM, HSNM, and WNM are read, hold, and write static noise margin, which is measured in milli volts (mV). The read delay time is measured in nano seconds (nS). P_Leak_, P_Read_, and P_Write_ are the leakage, read, and write power measured in nano watts (nW). The single SRAM bit cell area is measured in square micrometers (μm^2^). The EQM value of the SRAM cells calculated at the supply voltage of 1 V is plotted in Figure 40. The suggested E^2^VR11T SRAM cell’s overall quality metric is wider by 5.55×, 3.15×, 1.13×, and 0.23× against C6T, LP10T, MET11T, and S8T cells, respectively. The proposed E^2^VR11T SRAM is ideally a better choice for low-power applications considering its overall performance.

### 4.12. E^2^VR11T SRAM Overall Performance

The comparative results of various key parameters of all the cells together with proposed E^2^VR11T cell are summarized in Table 11. The simulation results shown in the table are measured at 1 V supply voltage and at 27 °C temperature with a transient time of 1 μs for all the cells. The variability investigations have been carried out by using MC simulation with 5000 samples and analyzed in detail to exhibit the resilience of the proposed cell. It can be easily inferred from the table that the proposed E^2^VR11T cell exhibits lower power, improved stability, higher speed with lesser delay time, better energy saving.

The variability analysis of the proposed cell in terms of power and delay time highlights lesser variability. The power variability demonstrates the process tolerance ability and delay time variability of presents the speed. Finally, the compact layout area and the larger electrical quality metric of the proposed cell compared to other cells absolutely exhibit the suitability of relevant memory array design and implementation.

## 5. Conclusions

In this research work, energy-efficient and variability-resilient 11T SRAM cell is presented and compared with other selected cells for power, current, delay, stability, and area overhead. A novel data-aware read–write assist technique is used to design the cell, which employs a dynamic differential approach for the write operation and single-ended read circuit for a read operation. The DARWA technique highly contributes to enhancing the read stability and write ability. The cell exhibits lower mean dynamic read power of 47.15% and 43.41% and an improved ready delay of 36% and 31% over the ST9T and LP10T cells. The write delay is also enhanced 16%, 40%, and 53% against S8T, LP10T, and MET11T cells in all process corners. The read energy is lower by 39%, 72%, and 59% than C6T, ST9T, and LP10T SRAM cells and lower write energies of 9%, 28%, 26%, and 52% against C6T, S8T, ST9T, and LP10T cells, respectively, which significantly denotes the energy efficiency. The cell achieves 1.94× and 0.18× higher read static noise margin compared to C6T and S8T cells and 8.70% and 19.57% enhancement of write noise margin over S8T and C6T cells.

The PVT variation results confirm that the proposed cell is energy efficient with significant process tolerance with variations. The proposed cell is also investigated for variability using Monte Carlo simulation on 5000 samples to find the statistical variation. The E^2^VR11T memory’s power variability of 0.0332 and 0.0356 and delay time variability of 0.0486 and 0.0381 for read and write operations confirms the variable resilience. The outcome of PVT variation analysis and variability investigation analysis validates the energy efficiency, robustness, and resilience of the proposed cell without any degradation. The cell demonstrates stable behavior at all supply voltages and at varying temperatures, which affirms that it is highly immune to process variation and environmental conditions.

The layout area of the E^2^VR11T cell is also substantially less than other cells. Finally, the electrical quality metric analysis outcome factor of 23.277 × 10^35^ justifies the overall performance of the proposed memory cell. In conclusion, the proposed E^2^VR11T SRAM cell design is an ideal choice and highly appropriate for reliable low-power applications that can be implemented at 45 nm nanoscale technology and beyond with the presence of PVT variation. Although the proposed E^2^VR11T cell has advantages in energy efficiency, power, performance, and variability resilience, it has some limitations. The limitations are that there is a bigger area occupancy when compared to a conventional 6T cell, and there is a larger number of peripheral circuits, which restrict its use for the implementation of larger cache size.

## Figures and Tables

**Table 1:** Operational specification of all the cells.

Cell Feature	C6T [20]	S8T [21]	ST9T [22]	LP10T [23]	MET11T [24]	E^2^VR11T (Proposed)
Write operation	Differential	Differential	Differential	Differential	Differential	Differential
Read operation	Differential	Single end	Differential	Differential	Single end	Single end
Bit lines *	2 BL/BLB	3 BL/BLB/RBL	1 BL	2 WBL/RBL	3 BL/BLB/RBL	3 BL/BLB/RBL
Control signals *	1 WL	2 WL/RWL	3 WL/WWLA/WWLB	2 WWL/RWL/WWLA	4 WWL/CWL/RWL/RGND	2 WL/RWL
No. of NMOS Transistors in Read path	-	2	-	-	2	3

* WL: word line; RWL: read word line; RBL: read bit line; WWL: write word line; CWL: column word line; RGND: read ground; BL, BLB: bit lines.

**Table 2:** Operational table of proposed E^2^VR11T SRAM cell.

Type	Signal	Write Operation	Read Operation	Hold State
Control Signals	WL	1	0	0
	RWL	0	1	0
	BL	1/0	Pre-charge	Pre-charge
Bit lines	BLB	0/1	Pre-charge	Pre-charge
	RBL	0	Pre-charge	Pre-charge

**Table 3:** Simulation parameters used in this work.

Parameters	Data
EDA design tool used	Cadence Virtuoso
CMOS technology	45 nm GPDK
Supply voltage	0.5–1.0 V
Temperature	27 °C
Device Size: PMOS	150 nm/45 nm
Device Size: NMOS	120 nm/45 nm

**Table 4:** Leakage power of E^2^VR11T SRAM cell.

SRAM Cell	Leakage Power (nW)	Variation
C6T [20]	70.4404	25.87%
S8T [21]	70.2984	25.72%
ST9T [22]	119.5427	56.32%
LP10T [22]	88.3565	40.90%
MET11T [23]	57.8335	9.71%
E^2^VR11T (proposed)	52.2160	-

**Table 5:** Read and write current of the cells.

SRAM Cell	Read Current (nA)	Variation	Write Current (nA)	Variation
C6T [20]	97.0769	12.81%	21.0656	−1.38%
S8T [21]	111.5110	24.09%	22.7555	6.15%
ST9T [22]	119.3510	29.08%	87.4934	75.59%
LP10T [22]	86.7418	2.42%	11.1768	−91.07%
MET11T [23]	112.8450	24.99%	10.0197	−113.13%
E^2^VR11T (proposed)	84.6452	-	21.3554	-

**Table 6:** Monte Carlo simulation of power variability analysis of cells (5000 samples).

No	SRAM Cells	Read Power			Write Power		
		Mean (nW)	Standard Deviation (nW)	Variability	Mean (nW)	Standard Deviation (nW)	Variability
		(μ)	(σ)	(σ/μ)	(μ)	(σ)	(σ/μ)
1	C6T [20]	70.81	1.677	0.0237	31.93	1.232	0.0386
2	S8T [21]	71.08	1.395	0.0196	34.32	1.856	0.0541
3	ST9T [22]	119.50	32.700	0.2736	107.70	31.100	0.2888
4	LP10T [22]	89.66	3.403	0.0380	36.99	2.070	0.0560
5	MET11T [23]	59.37	0.695	0.0117	14.55	0.670	0.0460
6	E^2^VR11T (proposed)	53.94	1.790	0.0332	29.47	1.049	0.0356

**Table 7:** Monte Carlo simulation of leakage power and current variability of cells (5000 samples).

No	SRAM Cells	Leakage Power			Leakage Current		
		Mean (nW)	Standard Deviation (nW)	Variability	Mean (nA)	Standard Deviation (nA)	Variability
		(μ)	(σ)	(σ/μ)	(μ)	(σ)	(σ/μ)
1	C6T [20]	70.40	1.679	0.0238	96.26	2.482	0.0258
2	S8T [21]	69.73	2.355	0.0338	108.30	4.180	0.0386
3	ST9T [22]	116.5	30.600	0.2627	105.50	30.200	0.2863
4	LP10T [22]	88.79	3.397	0.0383	85.30	5.637	0.0661
5	MET11T [23]	57.92	0.674	0.0116	111.70	1.763	0.0158
6	E^2^VR11T (proposed)	52.26	1.792	0.0343	82.44	5.146	0.0624

**Table 8:** Monte Carlo simulation of read, write current variability analysis of cells (5000 samples).

No	SRAM Cells	Read Current			Write Current		
		Mean (nA)	Standard Deviation (nA)	Variability	Mean (nA)	Standard Deviation (nA)	Variability
		(μ)	(σ)	(σ/μ)	(μ)	(σ)	(σ/μ)
1	C6T [20]	96.95	2.548	0.0263	18.01	0.877	0.0487
2	S8T [21]	111.60	1.783	0.0159	22.63	1.463	0.0647
3	ST9T [22]	110.70	30.810	0.2783	106.30	31.070	0.2923
4	LP10T [22]	86.99	5.671	0.0652	11.47	2.173	0.1896
5	MET11T [23]	113.00	1.764	0.0156	10.07	0.704	0.0699
6	E^2^VR11T (proposed)	84.41	5.167	0.0612	21.23	0.839	0.0395

**Table 9:** Monte Carlo simulation of read and write delay time variability of cells (5000 samples).

No	SRAM Cells	Read Delay Time (R_DT_)			Write Delay Time (W_DT_)		
		Mean (pS)	Standard Deviation (pS)	Variability	Mean (pS)	Standard Deviation (pS)	Variability
		(μ)	(σ)	(σ/μ)	(μ)	(σ)	(σ/μ)
1	C6T [20]	235.80	35.860	0.1521	343.30	13.030	0.0380
2	S8T [21]	101.00	15.650	0.1550	402.30	34.310	0.0853
3	ST9T [22]	119.50	32.700	0.2736	330.00	23.220	0.0704
4	LP10T [22]	274.40	11.500	0.0419	563.508	83.485	0.1482
5	MET11T [23]	100.90	0.002	-	725.20	37.490	0.0517
6	E^2^VR11T (proposed)	188.70	9.174	0.0486	336.90	12.850	0.0381

**Table 10:** Total power of 4 × 4 array.

Parameter	Data_0000	Data_1111
Total power	81.9196 nW	14.8521 μW
Total current	71.9991 nA	14.8412 μA

**Table 11:** Comparison of various parameters of all the cells.

	Parameters	C6T [20]	S8T [21]	ST9T [22]	LP10T [23]	MET11T [24]	E^2^VR11T (Proposed)
Power	Read power (nW)	70.716	71.023	120.765	89.639	59.224	53.839
	Write power (nW)	32.004	34.565	88.610	86.742	14.506	29.598
	Leakage power (nW)	70.440	70.298	119.543	88.357	57.834	52.216
Current	Read current (nA)	97.077	111.511	119.351	112.845	112.845	84.645
	Write current (nA)	21.066	22.756	87.493	11.177	10.019	21.355
	Leakage current (nA)	96.174	108.989	117.293	85.106	111.721	82.589
Stability	HSNM (mV)	400	400	400	400	400	420
	RSNM (mV)	160	400	440	400	420	470
	WNM (mV)	370	420	480	750	750	460
Speed	Read delay time (pS)	236.095	93.655	295.513	273.906	-	188.035
	Write delay time (pS)	343.658	402.153	152.056	560.399	722.505	336.972
Energy	Read Energy (aJ)	16.696	6.6516	35.688	24.553	-	10.126
	Write Energy (aJ)	10.999	13.901	13.474	20.686	10.481	9.974
Variability (σ/μ) on MC Simulation	Read power	0.0237	0.0196	0.2736	0.0380	0.0117	0.0332
	Write power	0.0386	0.0541	0.2888	0.0560	0.0460	0.0356
	Read delay time	0.0238	0.0338	0.2627	0.0383	0.0116	0.0343
	Write delay time	0.0258	0.0386	0.2863	0.0661	0.0158	0.0624
Area	Normalized	1	1.24	1.32	1.51	1.68	1.34
EQM	Electrical Quality Metric	3.555	18.944	-	5.615	10.939	23.277
	Supply voltage (V)	1	1	1	1	1	1

## Data Availability

Not applicable.
